# Biophysical Sensing Tools in Drug Discovery: Integrating Kinetics, Thermodynamics, Cellular Target Engagement and Structure

**DOI:** 10.3390/s26103105

**Published:** 2026-05-14

**Authors:** Esra Balıkçı, Caner Akıl

**Affiliations:** Division of Structural Biology, Nuffield Department of Medicine, University of Oxford, Oxford OX3 7BN, UK

**Keywords:** biophysical sensing, drug discovery, thermodynamics, SPR, NMR, mass spectrometry, target engagement, cryo-EM, X-ray crystallography, fragment-based drug discovery

## Abstract

Biophysical sensing technologies have become central to modern drug discovery because they enable direct, quantitative characterization of ligand–target interactions. In contrast to conventional biochemical and cellular assays that infer binding from downstream functional responses, biophysical methods detect interaction events through measurable physical changes such as refractive index, heat, fluorescence, mass, or protein stability. This review surveys the major classes of biophysical sensors used in drug discovery, including surface-based optical methods, calorimetry, solution-state spectroscopic techniques, mass spectrometry-based approaches, and cellular target engagement assays. For each modality, we outline the measurement principle, the key parameters obtained, and its value across hit identification, hit validation, lead optimization, and mechanism-of-action studies. We also emphasize the growing importance of combining orthogonal methods to improve confidence in binding data, resolve assay artifacts, and strengthen early decision-making. Finally, we discuss how biophysical measurements are increasingly integrated with structural biology and computational analysis to support more predictive and mechanism-driven discovery workflows. Collectively, these technologies provide a richer and more reliable understanding of molecular recognition and thereby improve the progression of drug candidates.

## 1. Introduction

Drug discovery fundamentally depends on the identification of molecules that bind biological targets with appropriate selectivity and favorable kinetic and thermodynamic properties [1]. Conventional biochemical and cellular assays remain indispensable in this process; however, they often provide only indirect evidence of binding because they rely on downstream functional or phenotypic outputs. This limitation is particularly relevant in early discovery, where assay interference, weak signals, and context-dependent effects can obscure the underlying mechanism of ligand–target recognition [2,3,4,5,6].

Biophysical sensing technologies address this limitation by measuring binding directly through physical changes such as refractive index, heat, fluorescence, mass, or thermal stability. Methods including surface plasmon resonance (SPR) [7], isothermal titration calorimetry (ITC) [8], nuclear magnetic resonance (NMR) [9], and mass spectrometry (MS) [10] can quantify equilibrium affinity, binding kinetics, stoichiometry, and thermodynamic parameters under label-free or minimally perturbed conditions. These measurements are increasingly recognized as important determinants of pharmacological performance, particularly as binding kinetics and residence time have emerged as critical complements to equilibrium affinity in compound optimization [7,11,12,13,14,15,16].

The relevance of biophysical methods has increased as drug discovery has moved toward more challenging target classes, including protein–protein interactions, intrinsically disordered proteins, and multicomponent assemblies. These systems frequently involve weak, transient, or conformationally heterogeneous interactions that are difficult to resolve using conventional assay formats. Consequently, biophysical methods now play a central role in early target validation, hit confirmation, and mechanism-guided optimization [6,17]. This is especially evident in fragment-based drug discovery, where weak initial binders are routinely identified and advanced using orthogonal methods such as NMR, SPR, and X-ray crystallography [4,17,18,19,20,21,22].

A major strength of current discovery workflows is the integration of complementary methods rather than reliance on a single assay platform [6]. Kinetic information obtained from SPR can be combined with thermodynamic data from ITC, structural and dynamic insight from NMR, and intracellular engagement data from assays such as CETSA and thermal proteome profiling (TPP). Such convergence improves confidence in binding assignments, helps distinguish genuine interactions from assay artifacts, and better connects in vitro measurements to cellular relevance [12,16,23,24,25,26].

Recent advances in native MS [10,27,28], hydrogen–deuterium exchange mass spectrometry (HDX-MS) [29], single-particle mass measurements [30,31], and computational analysis [32] have further expanded the scope of biophysical sensing. These approaches now support characterization of higher-order assemblies, conformational remodeling, and cooperative binding events that are increasingly important in degrader, molecular glue, and RNA-targeting discovery programs. As a result, biophysical sensing has evolved from a supporting analytical function into a core component of mechanism-driven drug discovery.

In this review, we summarize the major classes of biophysical sensing technologies used in drug discovery, outline the principal parameters they provide, and discuss their value across the discovery pipeline. We further examine how these methods are integrated with structural biology and emerging computational approaches to support more robust and predictive therapeutic development.

## 2. Biophysical Sensor Modalities: Principles, Readouts, and Roles in Drug Discovery

Biophysical sensing technologies convert physical changes associated with molecular recognition into measurable outputs. In contrast to functional assays, which report downstream biological consequences, these methods interrogate binding directly or capture structural, thermodynamic, and stability changes induced by ligand interaction [33]. Their practical value in drug discovery depends not only on the physical observable measured, but also on the stage of discovery at which the resulting information is most useful, including hit identification, hit confirmation, lead optimization, mechanistic characterization, and translational validation [12,13,22,26].

Different sensing modalities contribute different forms of insight. Surface-based optical methods are particularly effective for real-time kinetic analysis; calorimetric approaches define the energetic basis of binding; solution-state spectroscopic methods are well suited to weak, dynamic, or heterogeneous interactions; and mass-sensitive techniques are particularly informative for complex assemblies and conformational remodeling. Cellular target-engagement assays extend these measurements into more physiologically relevant environments. Together, these methods provide a multidimensional view of ligand–target recognition that is rarely achievable through any single platform alone [5,12,13,22,26]. A comparative overview of the principal sensor modalities, their signal types, key outputs, typical sensitivity ranges, and practical roles across discovery is provided in Table 1.

### 2.1. Surface-Based Optical Sensors (SPR, BLI)

Surface-based optical biosensors, most notably SPR and biolayer interferometry (BLI), detect binding by monitoring changes in refractive index or optical thickness near a sensor surface. In SPR, analyte binding to an immobilized ligand alters the local refractive index at a metal surface, producing a measurable shift in resonance conditions [7,12]. BLI operates through changes in light interference patterns generated by accumulation of biomolecular mass on the biosensor surface [34,65].

The principal advantage of these platforms is their ability to resolve binding kinetics in real time. By continuously monitoring association and dissociation phases, SPR and BLI can provide direct estimates of $k_{on}$, $k_{off}$, from which the equilibrium dissociation constant ($K_{D}$) can be derived [35]. This capability is especially valuable because compounds with similar equilibrium affinity may differ substantially in residence time, and such differences can influence target occupancy, pharmacodynamic duration, and in vivo efficacy [12,14,15,66,67,68].

SPR and BLI are widely applied in fragment screening, kinetic prioritization, competition studies, epitope binning, and antibody characterization. Improvements in instrumentation have increased both sensitivity and throughput, allowing analysis of weak interactions and supporting medium-throughput kinetic profiling in lead optimization [34,69,70]. Under optimized conditions, these methods can detect interactions across a broad affinity range, including weak fragment binding [4,12,65,71,72].

Despite these strengths, robust assay design remains essential. Protein immobilization may alter orientation, restrict conformational freedom, or reduce binding-site accessibility [11,12,18]. Mass transport limitations can distort kinetic fits, and non-specific surface interactions may generate misleading signals [35]. Accordingly, surface-based optical data are most reliable when supported by appropriate controls, optimized surface chemistry, rigorous referencing, and orthogonal validation in solution [11,12,18,73].

**Figure 1:**
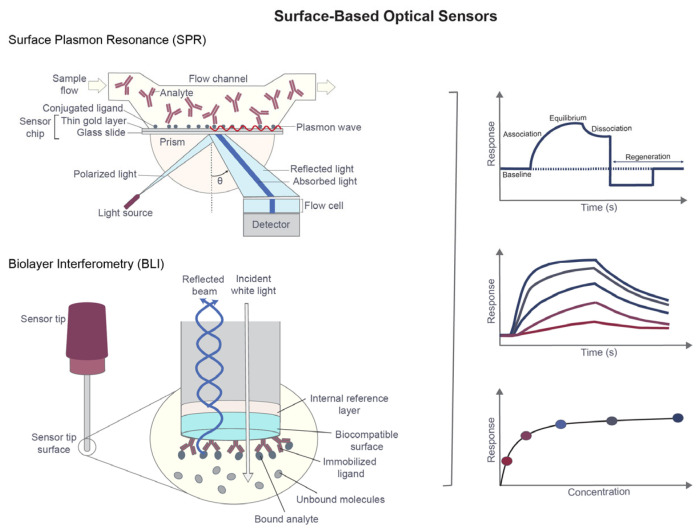
Surface-based optical sensors for real-time analysis of biomolecular interactions. SPR and BLI are label-free optical biosensing platforms widely used to characterize ligand–target interactions in drug discovery. In SPR, analyte binding to an immobilized ligand on a metal-coated sensor surface alters the local refractive index near the sensor interface, resulting in a shift in the SPR resonance angle (θ), which is detected through changes in reflected light intensity. SPR responses are typically reported in response units (RU), where the signal is proportional to the amount of material bound at the sensor surface. In BLI, biomolecular binding at the biosensor tip changes the optical interference pattern of reflected white light, producing a measurable wavelength shift. BLI responses are typically reported as shifts in optical thickness measured in nanometers (nm), which are proportional to the amount of biomolecular mass accumulated at the sensor surface. In both methods, the resulting sensorgrams contain association, equilibrium, dissociation, and regeneration phases that enable determination of kinetic and equilibrium binding parameters. Representative response curves illustrate concentration-dependent binding kinetics and equilibrium saturation behavior.

Overall, SPR and BLI are most powerful when kinetic information is central to compound prioritization (Figure 1). Their ability to distinguish compounds based on kinetic behavior, rather than equilibrium affinity alone, makes them particularly valuable in mechanism-resolved hit validation and lead selection [12,66,72].

### 2.2. Calorimetric Sensors (ITC, DSC)

Calorimetric methods quantify heat changes associated with molecular interactions and therefore provide direct insight into binding energetics. ITC measures the heat released or absorbed during ligand binding [13], whereas differential scanning calorimetry (DSC) monitors changes in protein thermal stability as temperature increases [74]. Because these methods do not require labels or immobilization, they are among the most direct approaches available for the study of biomolecular interactions [16].

ITC is particularly informative because it can provide a complete thermodynamic profile from a single experiment, including $K_{D}$, stoichiometry, enthalpy, entropy, and free energy. This level of characterization is highly relevant in medicinal chemistry, where similar affinity values may arise from different energetic contributions. Thermodynamic analysis can therefore clarify whether optimization reflects genuinely improved molecular recognition or merely compensatory energetic trade-offs [16,37,38,39,40]. Recent probe discovery against the bromodomain PHD finger transcription factor (BPTF) illustrates how ITC can support compound prioritization when integrated with structural and cellular target-engagement measurements [75].

The main limitations of ITC are lower throughput and relatively high material requirements compared with optical or fluorescence-based methods. For this reason, ITC is not typically used for primary screening, but it remains highly valuable for prioritized hits, lead series, and mechanistic confirmation. In these contexts, it provides a depth of energetic insight that is difficult to obtain by other means [37]. ITC has also been valuable outside classical medicinal chemistry workflows, for example in validating interaction specificity and stoichiometry in structurally divergent actin-regulatory systems [76].

DSC provides complementary information by detecting ligand-induced changes in protein thermal stability. Although it does not directly yield kinetic or affinity constants, it is useful for evaluating conformational stabilization, formulation effects, and protein integrity [74]. In drug discovery workflows, calorimetric methods are therefore best viewed as mechanistic tools that refine understanding of binding events rather than as high-throughput screening platforms.

**Figure 2:**
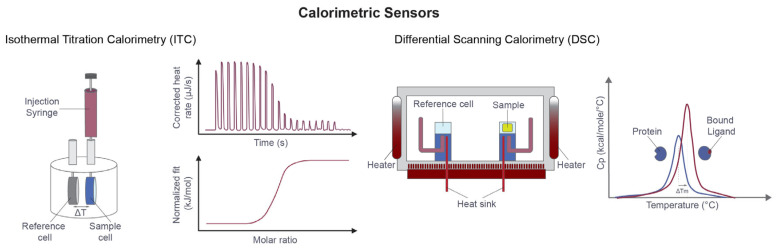
Calorimetric sensors for measuring the energetic consequences of ligand binding. In ITC, sequential ligand injections into a sample cell generate heat pulses that are integrated to produce a binding isotherm, from which affinity, stoichiometry, enthalpy, and entropy can be derived. In DSC, the difference in heat capacity between sample and reference cells is monitored during controlled heating, allowing detection of ligand-induced shifts in protein melting temperature and thermal stability. Representative DSC curves illustrate thermal stabilization upon ligand binding, where the blue curve represents the unbound protein and the red curve represents the ligand-bound state.

Together, calorimetric approaches are especially useful when the key question is not only whether a ligand binds, but also how the energetic basis of that interaction may guide compound optimization (Figure 2).

### 2.3. Solution-State Spectroscopic Sensors

**Figure 3:**
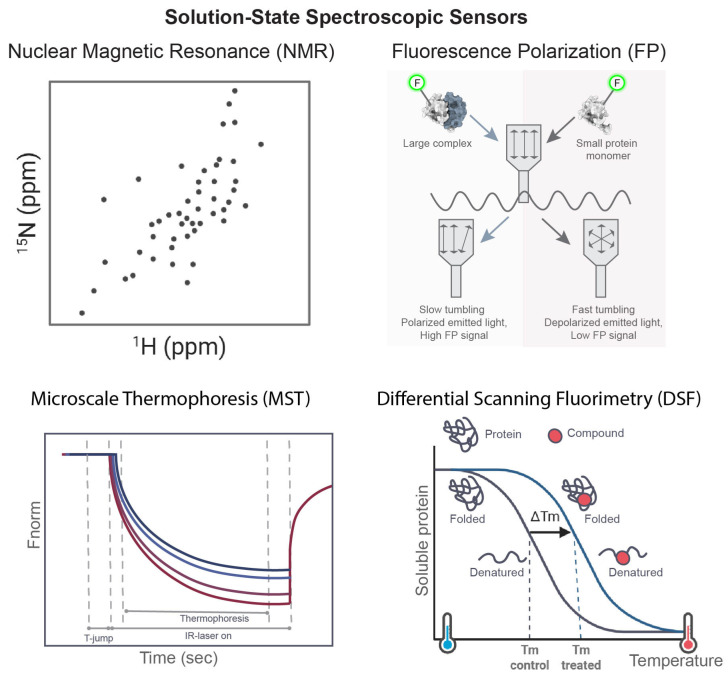
Representative solution-state spectroscopic sensors used in drug discovery include NMR, FP, MST, and DSF. NMR detects ligand-induced chemical shift changes and provides direct information on binding and molecular environment in solution. FP monitors changes in rotational diffusion of a fluorescent probe (F, shown in green), enabling detection of binding or competitive displacement through polarization changes. MST measures altered thermophoretic behavior upon complex formation and is widely used to determine equilibrium affinity in free solution. DSF detects ligand-induced thermal stabilization by monitoring protein unfolding during heating.

Solution-state spectroscopic methods examine interactions in free solution rather than on immobilized surfaces (Figure 3). This is an important practical advantage because it reduces the likelihood of surface-related artifacts and better preserves native conformational equilibria. These methods are particularly useful for weak binders, dynamic systems, and targets in which conformational heterogeneity is mechanistically important [41,42].

#### 2.3.1. Nuclear Magnetic Resonance (NMR)

NMR is among the most information-rich techniques used in drug discovery. Ligand binding perturbs the magnetic environment of atomic nuclei, leading to measurable changes in chemical shifts, relaxation rates, or signal intensity. Depending on the experiment employed, these perturbations can reveal whether binding occurs, where it occurs, and how it influences molecular dynamics [41,43,44].

Ligand-observed experiments, such as STD-NMR [45] and WaterLOGSY [46], are particularly useful in fragment-based drug discovery because they can detect weak interactions in the high micromolar to millimolar range and generally do not require isotopically labeled protein. Protein-observed approaches, especially HSQC-based methods, provide residue-level information and can directly map binding interfaces. These experiments can also distinguish orthosteric from allosteric interactions and provide insight into induced fit, conformational selection, and binding-induced ordering of flexible regions [4,41,42,43,44].

NMR is especially valuable for dynamic and difficult targets, including intrinsically disordered proteins and conformationally flexible domains, for which static structural approaches may be less informative. Although throughput is lower than that of many screening-oriented methods and isotope labeling may increase experimental complexity, NMR remains one of the most powerful orthogonal tools for hit validation, binding-site mapping, and mechanistic interpretation [20].

#### 2.3.2. Microscale Thermophoresis (MST)

MST measures the movement of molecules in microscopic temperature gradients. Ligand binding alters properties such as molecular size, charge distribution, hydration shell, or conformation, which in turn affect thermophoretic behavior [47]. By monitoring fluorescence during titration of one interaction partner, MST generates equilibrium binding curves from which $K_{D}$ values can be obtained [47,48,49].

A major advantage of MST is that it is performed in free solution and is compatible with a broad range of buffer compositions, detergents, and complex sample environments. It also requires relatively small amounts of material and can cover a wide affinity range [47,48,49]. In practice, MST is often used as an orthogonal validation method after initial screening and as a medium-throughput affinity assay during hit expansion and lead optimization. Because MST combines operational flexibility with quantitative output, it occupies a useful position between rapid screening methods and more detailed structural or mechanistic techniques [47,48,49,50].

#### 2.3.3. Differential Scanning Fluorimetry (DSF)

DSF, also referred to as thermal shift analysis, detects ligand-induced changes in protein stability during controlled heating [51]. As proteins unfold, exposed hydrophobic regions interact with an environmentally sensitive fluorescent dye, allowing estimation of melting temperature. Ligand binding frequently stabilizes the protein and results in an upward thermal shift [51,52].

The method is simple, inexpensive, and scalable, which makes it particularly useful for hit confirmation and triage. It is well suited to early-stage campaigns in which large numbers of compounds must be evaluated rapidly and where a stability-based readout is sufficient for prioritization [51,53].

However, DSF does not directly provide equilibrium or kinetic binding parameters, and thermal shifts do not necessarily correlate closely with affinity. Interpretation may also be complicated by aggregation, destabilizing ligands, or buffer-dependent effects on protein stability. Consequently, DSF is most effective as a rapid prioritization tool rather than as a stand-alone demonstration of binding.

#### 2.3.4. Fluorescence Polarization (FP)

FP measures changes in rotational diffusion of a fluorescently labeled ligand. When a small fluorescent probe binds to a larger protein, rotational mobility decreases, producing an increase in the polarization of emitted light. In drug discovery, this principle is widely used in competitive displacement assays to evaluate ligand binding and inhibitor potency [54].

Because FP is highly compatible with multiwell plate formats, it remains an important method for high-throughput screening and structure–activity relationship studies [55]. It is particularly useful when a robust fluorescent probe is available and when competitive binding is the intended readout. Its principal limitations arise from dependence on labeled probes, possible compound fluorescence interference, and lower mechanistic resolution relative to direct biophysical methods [54,55,56]. Nonetheless, FP remains a practical and efficient assay format in many discovery programs.

### 2.4. MS-Based and Single-Particle Mass Sensors

**Figure 4:**
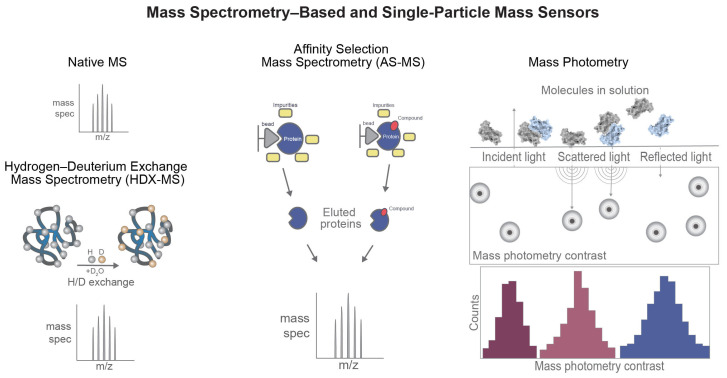
Mass-sensitive sensing platforms expand the range of measurable drug–target interactions beyond simple binary binding. Native MS preserves intact noncovalent assemblies and enables direct determination of binding stoichiometry and higher-order complex formation. HDX-MS reports ligand-induced protection patterns and conformational remodeling by monitoring deuterium incorporation across the protein. AS-MS identifies binders from compound mixtures after target-based enrichment, supporting label-free screening and hit identification. Mass photometry detects individual particles in solution through light-scattering contrast and yields mass distributions for heterogeneous populations and oligomeric states.

MS-based sensors detect biomolecular interactions by measuring mass-to-charge ratios under conditions that preserve noncovalent complexes or report on structural changes induced by ligand binding (Figure 4). In drug discovery, these techniques are particularly valuable in systems where stoichiometry, cooperativity, multicomponent assembly, or conformational remodeling are central to mechanism [10,27,28].

#### 2.4.1. Native MS

Native MS preserves protein–ligand and protein–protein complexes in near-native states during ionization, typically using gentle electrospray conditions and volatile buffers [27]. Because intact complexes can be transferred into the gas phase without complete dissociation, native MS enables direct observation of bound assemblies and accurate determination of stoichiometry, including higher-order complexes [27,28].

This capability is particularly important for multimeric targets, membrane proteins stabilized in nanodiscs or detergents, and emerging therapeutic modalities such as Proteolysis-Targeting Chimera (PROTAC) degraders that rely on cooperative ternary complex formation between a target protein, a small molecule, and an E3 ligase. By resolving multiple charge states and complex compositions within a single spectrum, native MS can quantify binding populations, detect cooperativity, and distinguish between specific complex formation and nonspecific aggregation [10,27,28]. As a result, it provides a powerful orthogonal validation tool for confirming interaction mechanisms.

#### 2.4.2. Affinity Selection MS (AS-MS)

AS-MS combines equilibrium binding with mass-based detection. In this approach, compound mixtures are incubated with a target protein, and bound ligands are separated from unbound species using techniques such as ultrafiltration, size exclusion, or immobilized target capture. The retained ligands are then identified by MS [77].

AS-MS supports medium-throughput screening without the need for fluorescent or radioactive labeling and is particularly well suited for fragment screening campaigns. Because detection is based on molecular mass rather than reporter signals, AS-MS is less susceptible to common assay artifacts, including compound fluorescence, aggregation, or redox interference [77,78]. This makes it a robust method for early-stage hit identification and validation.

#### 2.4.3. Hydrogen–Deuterium Exchange MS (HDX-MS)

HDX-MS provides complementary structural information by measuring backbone amide hydrogen exchange rates in the presence and absence of ligand. Upon ligand binding, regions of the protein that become stabilized or shielded from solvent show reduced deuterium incorporation [29,57]. By combining proteolytic digestion with peptide-level analysis, HDX-MS can map conformational changes, identify protected binding regions, and reveal allosteric communication pathways across the protein structure [29,57,58]. This technique is particularly valuable for distinguishing between induced fit and conformational selection mechanisms and for studying dynamic systems that are difficult to capture using crystallography.

In drug discovery, HDX-MS is widely used for epitope mapping, validation of allosteric modulators, and support of structure-guided optimization, especially in cases where high-resolution structural data are unavailable.

#### 2.4.4. Mass Photometry

Mass photometry is an emerging single-particle technique that measures biomolecular mass in solution by detecting interferometric light scattering from individual particles transiently interacting with a glass surface. Because scattering intensity scales with molecular mass, the method enables direct analysis of proteins, nucleic acids, and ligand-induced complexes under near-native conditions [30]. Unlike ensemble-based methods, mass photometry resolves heterogeneous populations at the single-particle level [30,79].

In drug discovery, mass photometry is particularly useful for studying oligomerization, stoichiometry, and assembly formation. The technique can distinguish monomers, dimers, higher-order oligomers, and ligand-stabilized complexes without requiring labeling or immobilization [80]. This is especially relevant for protein–protein interaction targets, molecular glues, and targeted protein degradation systems involving ternary complex formation [31,79].

A major advantage of mass photometry is its ability to quantify binding populations by molecular counting, thereby providing information on apparent stoichiometry and complex formation in solution [31]. The method also offers low sample consumption, rapid analysis, and compatibility with heterogeneous systems [80]. However, it does not directly provide structural or kinetic parameters and is therefore most informative when combined with complementary methods such as native MS, SPR/BLI, HDX-MS, cryo-EM, or X-ray crystallography.

### 2.5. Cellular and Systems-Level Sensors

In vitro interaction data are informative only to the extent that they translate into target engagement in a cellular environment. Cellular and systems-level assays address this limitation by examining compound action in the context of permeability, subcellular localization, endogenous interaction partners, and intracellular state [24,25,26]. They therefore provide a critical bridge between purified-protein measurements and biological relevance. Recent off-target profiling of clinical BTK inhibitors against NUDT5 further illustrates how orthogonal biophysical, structural, and cellular engagement assays can uncover liabilities that might be missed by pathway-centric or purely biochemical readouts [81].

#### 2.5.1. The Cellular Thermal Shift Assay (CETSA)

CETSA is based on the principle that ligand binding often stabilizes proteins against thermal denaturation. Following compound treatment and heat challenge, the soluble fraction of the target protein is quantified; a shift in thermal stability is interpreted as evidence of intracellular target engagement. Because the assay can be performed in intact cellular systems without engineered labeling of the target, it provides a direct readout of binding in a more physiologically relevant context [59].

CETSA is especially useful because it addresses a critical translational question that purified-protein assays cannot answer independently: whether a compound engages its intended target inside cells [60]. It is therefore widely used to confirm intracellular engagement, compare compounds within a chemical series, and relate biochemical potency to cellular activity [59,60].

#### 2.5.2. Thermal Proteome Profiling (TPP)

TPP extends the CETSA concept to the proteome scale by coupling thermal stability profiling with quantitative MS. This allows simultaneous assessment of target engagement and off-target stabilization across large portions of the proteome [61,62].

The principal value of TPP lies in its systems-level perspective. It can identify unexpected target classes, reveal pathway-level responses, and detect broader proteome remodeling associated with compound treatment [61,62]. This is particularly useful during early development, where off-target effects may confound phenotypic observations or contribute to toxicity risk. However, TPP also introduces substantial analytical complexity and requires careful normalization, curve fitting, and statistical control [61,62,63,64].

#### 2.5.3. Label-Free Impedance-Based Cellular Sensing

Impedance-based systems monitor electrical impedance across cells grown on microelectrodes [82]. Because impedance changes with cell adhesion, morphology, proliferation, migration, and barrier function, these platforms provide real-time, label-free measurements of integrated cellular responses to compound treatment [82,83,84]. Although impedance does not provide a direct measure of target engagement, it is useful for monitoring the dynamic cellular consequences of ligand action, particularly for receptors, ion channels, and signaling pathways that rapidly alter cell shape or adhesion [83,85]. Its principal limitation is interpretability, since the signal reflects multiple cellular processes simultaneously. For this reason, impedance-based measurements are most informative when interpreted alongside more direct molecular or target-engagement assays [83,84,85].

**Figure 5:**
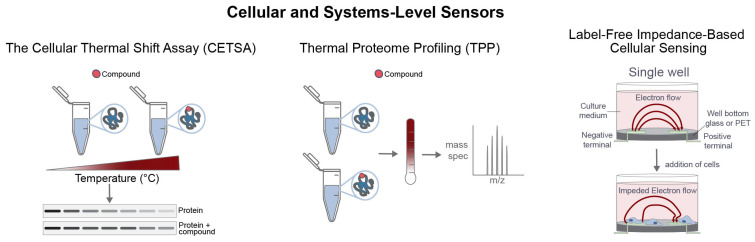
Cellular biophysical sensors extend interaction measurements into biologically relevant environments. The CETSA detects intracellular target engagement by measuring ligand-induced stabilization of proteins against heat-induced aggregation. TPP applies the same principle at proteome scale by combining thermal denaturation with MS-based quantification, enabling simultaneous assessment of on-target and off-target effects. Label-free impedance-based cellular sensing monitors changes in electron flow across electrode-containing wells as a function of cell adhesion, morphology, growth, and response to compound treatment.

Taken together, CETSA, TPP, and label-free cellular response platforms reduce translational uncertainty by linking molecular interaction data to intracellular engagement and systems-level response (Figure 5). When integrated with upstream biophysical measurements, they provide a stronger basis for progression decisions in drug discovery.

## 3. Complementarity with X-Ray Crystallography and Cryo-EM

Structural biology is not a substitute for biophysical sensing, but it is one of its most important complements. Biophysical techniques establish whether molecules bind, how strongly they bind, how rapidly they associate and dissociate, and whether they alter stability or conformational dynamics [20]. Structural biology complements biophysical sensing by defining where binding occurs and how conformational states are stabilized and visualized at atomic or near-atomic resolution [20,86]. The combination of these approaches is central to mechanism-guided drug discovery [20,22,86].

### 3.1. Binding Mode Validation

One of the most practical contributions of X-ray crystallography and cryo-electron microscopy (cryo-EM) is binding-mode validation [86]. Kinetic or thermodynamic measurements can confirm a productive interaction, but they cannot directly reveal ligand orientation, local contact networks, water-mediated interactions, or induced pocket formation. Structural methods resolve these questions and thereby reduce ambiguity in medicinal chemistry decisions [2,5,6,20,22].

This complementarity is particularly important in fragment-based drug discovery, where weak initial binders must be optimized using reliable structural information [87]. Once fragment hits are confirmed by orthogonal biophysical methods, co-crystallography can reveal their exact positioning within a binding pocket and guide fragment growing, merging, or linking strategies [88,89]. Structural validation is also critical in allosteric discovery programs, where confirming engagement of a regulatory site rather than the orthosteric site may determine the entire optimization strategy [4,90,91].

The development of KRAS (G12C) inhibitors illustrates the importance of this integration [92,93]. Biophysical and functional data established productive engagement, while structural analysis revealed occupancy of a previously underappreciated pocket and clarified the conformational basis of selective inhibition [94]. More broadly, structural confirmation helps ensure that improvements in affinity reflect productive and specific interactions rather than assay artifacts or suboptimal binding modes.

### 3.2. Conformational Plasticity

Proteins frequently exist as dynamic ensembles rather than single rigid structures, and structural biology is essential for defining which conformational states are stabilized by different ligands. This is especially relevant for kinases, G protein-coupled receptors, molecular machines, and intrinsically disordered or partially ordered systems [95,96,97].

In such cases, biophysical data may reveal differences in affinity, kinetics, or target engagement, whereas structural data provide the mechanistic explanation by showing how ligands stabilize distinct conformational states. X-ray crystallography has long been central to this type of analysis, and cryo-EM has substantially expanded access to large or flexible assemblies that are difficult to crystallize [98]. In GPCR discovery, for example, cryo-EM has clarified how ligands stabilize distinct signaling states and thereby linked conformational selection to pharmacological output [99]. More recently, in antiviral research, in situ cryo-ET analysis of SARS-CoV-2 fusion has resolved transient conformational trajectories in a near-native setting and provided mechanistic insight into antibody binding during membrane fusion [100]. Similar structural approaches have also helped refine understanding of the relationship between HIV-1 maturation and infectivity [101].

For targeted protein degradation and molecular glue strategies, conformational plasticity is often directly related to efficacy [102,103]. Biophysical assays can establish ternary complex formation and cooperativity, but structural methods clarify the geometry, interface compatibility, and conformational constraints required for productive ubiquitination or induced protein association [102,103,104,105].

### 3.3. Multicomponent Complexes

The growing complexity of therapeutic modalities has increased the importance of structural methods for resolving multicomponent assemblies. While traditional inhibitors often involve binary target-ligand interactions, many contemporary modalities depend on ternary or higher-order complexes [20,106].

Cryo-EM is especially valuable for large assemblies such as membrane protein super complexes, transcriptional machines, and degrader-induced ternary systems [107,108]. X-ray crystallography remains highly informative when crystals can be obtained, particularly for enzyme–cofactor–ligand systems or small-molecule modulators of protein–protein interfaces [109]. In both cases, structural data prevent misinterpretation of biophysical readouts by showing whether an observed interaction corresponds to a biologically meaningful binding mode [75,81].

**Figure 6:**
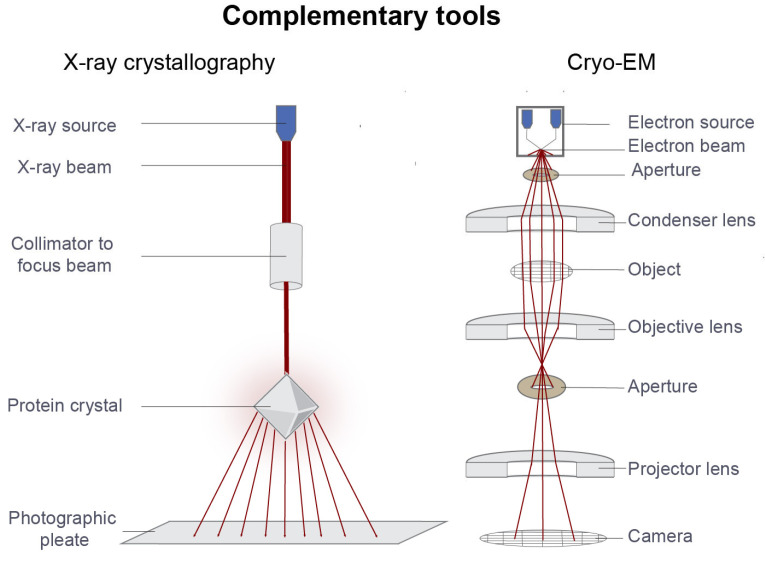
Complementary structural tools that contextualize biophysical sensor readouts. X-ray crystallography and cryo-EM complement biophysical sensing by providing structural information on ligand binding and macromolecular architecture. X-ray crystallography determines atomic details of ligand placement and local interaction networks in diffraction-quality crystals, whereas cryo-EM resolves larger or more conformationally heterogeneous assemblies without requiring crystallization. Although these approaches are not binding sensors in the same sense as kinetic, calorimetric, or cellular platforms, they provide the structural framework needed to interpret sensor-derived measurements of affinity, kinetics, stability, and conformational change.

Taken together, X-ray crystallography and cryo-EM do not replace biophysical sensors (Figure 6); rather, they provide the structural framework that makes biophysical measurements more mechanistically interpretable and more actionable for molecular design.

## 4. Emerging Technologies and Future Perspectives

Biophysical sensing is evolving in response to increasingly difficult targets, more complex therapeutic modalities, and the demand for earlier translational confidence. Future discovery workflows are likely to rely on sensing platforms that are more integrated, more miniaturized, and more computationally connected than those used in conventional pipelines.

### 4.1. Miniaturization and High Throughput

One major trend is the progressive miniaturization and parallelization of established methods. Historically, detailed kinetic and thermodynamic analyses were often constrained by low throughput and substantial material requirements. Advances in microfluidics, automation, and sample handling are now reducing these barriers and enabling quantitative biophysical measurements earlier in discovery.

This trend is especially evident in SPR, MST, and MS-based workflows, where improved fluidics, multiplexing, and automated preparation are increasing experimental throughput while preserving mechanistic depth [7,10,48]. As a result, parameters such as residence time, stoichiometry, and cooperative assembly are becoming more accessible during early hit triage and lead optimization [10,47,49,65,67].

### 4.2. Single-Molecule and Real-Time Intracellular Sensors

A second major direction is the shift toward higher-resolution measurements in living systems and at the single-molecule level [110,111]. Single-molecule force spectroscopy approaches, including atomic force microscopy and optical tweezers, can directly probe interaction forces, dissociation pathways, and intermediate states. These measurements reveal features of energy landscapes that are frequently obscured in ensemble experiments [110,111].

In parallel, live-cell sensing methods based on fluorescence resonance energy transfer (FRET) [112,113], bioluminescence resonance energy transfer (BRET) [114,115], and related approaches are expanding the ability to monitor ligand–target interactions in real time within native cellular environments. These techniques provide temporal resolution that is difficult to achieve with endpoint assays and may be particularly useful for transient complexes, rapid signaling systems, and context-dependent target engagement.

### 4.3. Expanding Therapeutic Modalities

The diversification of therapeutic strategies is placing new demands on sensing technologies. Modalities such as PROTACs, molecular glues, RNA-targeting ligands, and compounds directed at intrinsically disordered proteins require methods capable of measuring cooperativity, induced proximity, multivalency, and structural remodeling, rather than simple binary binding alone [102,116,117,118,119,120,121,122,123]. Recent degrader studies targeting NUDT5 further illustrate how targeted protein degradation workflows increasingly depend on integrated target-engagement, ternary-complex, and phenotypic measurements to disentangle enzymatic from non-enzymatic target functions [105].

This shift is already reshaping biophysical workflow design. Increasingly, discovery programs must combine kinetic, structural, conformational, and cellular measurements to capture the behavior of compounds that act through assembly formation or dynamic state selection. As these modalities mature, methods that can simultaneously address mechanistic complexity and physiological relevance will become even more important.

### 4.4. AI-Integrated Sensing Platforms

As biophysical datasets become larger and more multidimensional, computational integration is becoming a practical necessity. Machine learning approaches are increasingly being applied to kinetic, thermodynamic, structural, and cellular datasets in order to prioritize compounds, detect hidden relationships, and support adaptive experimental design [2,124,125,126,127,128,129].

These approaches are particularly promising when integrated with proteomics-scale methods such as TPP or with multimodal datasets spanning binding, conformational change, and phenotypic response [126,130]. Their utility, however, will depend not only on algorithmic sophistication but also on the quality, reproducibility, and standardization of the experimental inputs [131]. Importantly, the development of predictive machine learning models will also require well-curated negative datasets, including compounds that fail to bind or produce measurable target engagement under standardized conditions. Such negative data are particularly important for distinguishing true molecular recognition from nonspecific aggregation, fluorescence interference, unstable ternary-complex formation, or context-dependent cellular effects that may otherwise confound model training. Inclusion of experimentally validated nonbinders and inactive analogs can substantially improve model calibration, reduce false-positive predictions, and enhance the generalizability of AI-driven approaches across chemically and mechanistically diverse datasets [132]. In this sense, the future impact of AI in biophysical sensing is tightly linked to advances in assay harmonization, data interoperability, and standardized reporting practices [131].

## 5. Discussion

Biophysical sensing technologies have become indispensable in modern drug discovery by providing direct, mechanistically informative views of ligand–target interactions. Their value extends beyond simple binding detection; they resolve critical dimensions such as affinity, kinetics, thermodynamics, stoichiometry, and conformational changes. This multidimensional perspective is particularly vital in early discovery to distinguish true molecular interactions from the indirect or artefactual readouts common in conventional assays [11,12,13,65].

As summarized in Table 1, every technique introduces distinct experimental assumptions and interpretive constraints. In addition, the practical sensitivity and lower detection capabilities of biophysical methods can vary substantially depending on assay format, target system, ligand properties, and instrumentation. Consequently, the most robust discovery workflows rely on orthogonal combinations of methods [73,133]. It is important to note that orthogonality does not always imply concordance. For instance, a compound might show measurable binding via SPR but fail to produce a thermal shift in DSF, not necessarily due to assay failure, but because the interaction may not stabilize the protein fold or may involve weak binding enthalpy [6,11]. Similarly, cellular target-engagement assays may not reproduce affinities observed in purified systems because of permeability, protein-complex formation, or intracellular competition effects [59,60]. Rather than viewing these discrepancies as errors, they should be interpreted mechanistically to distinguish genuine binders from non-specific interactions, aggregation artifacts, or context-dependent binding events [6,11].

The inherent analytical limitations of each technique further necessitate this integrated approach. Surface-based methods (SPR, BLI) are prone to immobilization artifacts and mass-transport effects [7,65,134], while fluorescence-based assays (FP, FRET, DSF) often suffer from signal interference and compound autofluorescence [55,135]. Even “gold standard” techniques have trade-offs: ITC provides unparalleled thermodynamic detail but requires high material consumption, and may fail to resolve interactions with weak enthalpic contributions [13,134], while structural methods (X-ray, Cryo-EM) provide atomic resolution but often capture static states that may not fully reflect solution or cellular dynamics [86,107]. Similarly, cellular assays such as CETSA and TPP improve physiological relevance but may complicate mechanistic interpretation because target engagement can be influenced by permeability, protein-complex formation, cellular localization, and indirect pathway effects [59,62]. These analytical constraints, coupled with practical hurdles such as scalability and resource intensity, dictate the architecture of modern drug discovery [73,134]. Consequently, the field has moved toward tiered workflows, where efficient, high-throughput assays (e.g., DSF, FP) serve as early triage tools, followed by progressively more rigorous biophysical and cellular analyses (e.g., NMR, HDX-MS, CETSA) to validate prioritized compounds.

Furthermore, the choice of molecular recognition elements (MREs), from highly specific antibodies to robust but less selective synthetic polymers, adds another layer of complexity to assay specificity [136]. Antibodies and engineered binding proteins often provide high target specificity and sensitivity in complex biological samples, whereas enzymes can offer functional selectivity linked to catalytic activity [136,137]. Synthetic recognition systems such as molecularly imprinted polymers may improve robustness and chemical stability but often provide lower selectivity than biologically evolved binders [138,139]. In practice, assay specificity is determined not only by the intrinsic affinity of the recognition element, but also by assay format, immobilization strategy, buffer composition, and the conformational state of the target protein [139]. Consequently, reliable data interpretation requires a holistic view that integrates assay-specific controls with complementary methodologies [134,135].

The need for such integration becomes even more pronounced as discovery programs shift toward more difficult targets and more complex modalities. Weak interactions, transient complexes, conformational ensembles, and cooperative multicomponent assemblies often cannot be understood adequately through equilibrium affinity alone [18,19,20,28,31]. In these scenarios, convergent evidence from multiple platforms is a prerequisite for reliable decision-making. To manage this complexity, effective strategies employ tiered workflows: high-throughput assays (DSF, FP) are used for early triage, followed by information-rich but lower-throughput methods (NMR, ITC, HDX-MS) for prioritized leads.

Ultimately, there is a growing necessity to translate these biophysical insights across biological scales. Measuring binding against purified proteins is no longer sufficient; intracellular engagement and proteomic context must be incorporated earlier in the pipeline. Assays like CETSA and TPP bridge this gap, connecting molecular recognition with biological relevance before costly downstream development [59,60,140,141]. This shift is supported by an expanding open-science infrastructure, providing the standardized probes and benchmarking datasets necessary for cross-platform validation [142].

Looking ahead, advances in miniaturization, single-molecule analysis, and computational integration are likely to strengthen the role of biophysical sensing even further. These developments may enable more predictive coupling of interaction data with biological behavior and improve the early identification of compounds with favorable translational profiles. In this context, biophysical sensing is best viewed not as a collection of specialized analytical tools, but as a central decision-making layer that connects molecular mechanism to therapeutic progression.

## 6. Conclusions

Biophysical sensing technologies are now central to contemporary drug discovery because they enable direct and quantitative characterization of ligand–target interactions across multiple mechanistic dimensions. By measuring affinity, kinetics, thermodynamics, stoichiometry, conformational effects, and cellular target engagement, these methods reduce uncertainty during hit validation and lead optimization and support more informed progression decisions.

Their greatest value emerges when they are used in combination. The integration of surface-based, solution-state, mass-sensitive, structural, and cellular approaches provides a more reliable and mechanistically coherent picture of molecular recognition than any single assay alone. As miniaturization, data standardization, and computational integration continue to advance, biophysical sensing will play an even more important role in predictive, mechanism-driven drug discovery workflows.

## Figures and Tables

**Table 1:** Comparative overview of major biophysical sensing modalities used in drug discovery. Signal type, principal readouts, typical sensitivity range, representative limits of detection (LOD), typical material consumption, key limitations, and representative references (Refs) are summarized for the indicated biophysical sensing tools.

Tool	Signal Type	Extracted Parameters	Sensitivity	Representative LOD *	Typical Material Consumption	Key Limitations	Refs
SPR/BLI	Refractive index/interference	K_D_, k_on_, k_off_	pM–mM	SPR: ~1–10 pg/mm^2^ surface mass; BLI: sub-nM–nM depending on format	Low (ng–µg protein; low µL volumes)	Surface immobilization artifacts; mass transport effects; non-specific binding; high cost	[7,12,34,35]
ITC	Heat	ΔH, ΔS, K_D_, n	nM–µM	Typically low nM affinity range under optimized conditions	High (10–100 µg protein per experiment; high µM concentrations)	High material consumption; low throughput; requires measurable enthalpy	[13,36,37,38,39,40]
NMR	Chemical shift	Binding site, K_D_, dynamics	µM–mM	Typically high µM–mM depending on experiment and protein size	Medium–High (0.1–1 mM protein; hundreds of µL)	High sample concentration; low throughput; size limitations	[9,41,42,43,44,45,46]
MST	Thermophoresis	K_D_	pM–mM	Low pM–nM under optimized fluorescence conditions	Low (nM–µM; few µL)	Requires labeling or intrinsic fluorescence; sensitive to buffer conditions	[47,48,49,50]
DSF/TSA	Thermal stability	ΔTm	µM	Typically low µM ligand stabilization detectable	Low–Medium (µg protein; small volumes)	Indirect binding readout; not all interactions shift Tm	[51,52,53]
FP	Fluorescence polarization	IC_50_, K_D_	nM–µM	Typically low nM fluorescence detection	Low (nM ligand; small volumes)	Requires labeling; susceptible to fluorescence interference	[54,55,56]
Native MS	Mass/charge	Stoichiometry, complexes	nM–µM	fM–pM ion detection; practical binding range nM–µM	Low–Medium (µg protein)	Buffer constraints; limited for weak/transient complexes	[10,27,28]
HDX-MS	Isotope exchange	Conformational changes, interfaces	nM–µM	Peptide-level detection in low nM range	Medium (µg–mg protein)	Complex data analysis; moderate resolution	[29,57,58]
CETSA/TPP	Thermal stability (cellular)	Target engagement	µM	Typically µM cellular engagement window	Medium–High (cells or lysate; mg protein equivalent)	Indirect readout; requires orthogonal validation; limited kinetic insight	[59,60,61,62,63,64]

* Representative LOD values are approximate and may vary substantially depending on assay format, instrumentation, target system, labeling strategy, immobilization conditions, and experimental setup.

## Data Availability

No new data were created or analyzed in this study. Data sharing is not applicable to this article.

## Abbreviations

The following abbreviations are used in this manuscript:

AS-MS	Affinity Selection Mass Spectrometry
BLI	Biolayer Interferometry
BRET	Bioluminescence Resonance Energy Transfer
CETSA	Cellular Thermal Shift Assay
cryo-EM	Cryo-Electron Microscopy
DSC	Differential Scanning Calorimetry
DSF	Differential Scanning Fluorimetry
FP	Fluorescence Polarization
FRET	Fluorescence Resonance Energy Transfer
GPCR	G Protein-Coupled Receptor
HDX-MS	Hydrogen–Deuterium Exchange Mass Spectrometry
HSQC	Heteronuclear Single Quantum Coherence
WaterLOGSY	Water-Ligand Observed via Gradient SpectroscopY
ITC	Isothermal Titration Calorimetry
K_D_	Equilibrium Dissociation Constant
k_off_	Dissociation Rate Constant
k_on_	Association Rate Constant
MS	Mass Spectrometry
MST	Microscale Thermophoresis
MRE	Molecular Recognition Elements
NMR	Nuclear Magnetic Resonance
PROTAC	Proteolysis-Targeting Chimera
SPR	Surface Plasmon Resonance
STD-NMR	Saturation Transfer Difference Nuclear Magnetic Resonance
TPP	Thermal Proteome Profiling
Tm	Melting Temperature
